# Power Law for Estimating Underdetection of Foodborne Disease Outbreaks, United States 

**DOI:** 10.3201/eid3002.230342

**Published:** 2024-02

**Authors:** Laura Ford, Julie L. Self, Karen K. Wong, Robert M. Hoekstra, Robert V. Tauxe, Erica Billig Rose, Beau B. Bruce

**Affiliations:** Centers for Disease Control and Prevention, Atlanta, Georgia, USA

**Keywords:** foodborne outbreaks, disease outbreaks, foodborne diseases, food safety, statistical distributions, public health surveillance, United States

## Abstract

We fit a power law distribution to US foodborne disease outbreaks to assess underdetection and underreporting. We predicted that 788 fewer than expected small outbreaks were identified annually during 1998–2017 and 365 fewer during 2018–2019, after whole-genome sequencing was implemented. Power law can help assess effectiveness of public health interventions.

Each year in the United States, >800 foodborne outbreaks are reported, causing >14,000 illnesses and >800 hospitalizations (*1*–*3*). Foodborne outbreaks range from small, localized outbreaks, such as those associated with a locally contaminated meal shared by family or friends, to large, multistate outbreaks associated with a contaminated food that is widely distributed. Selection and information biases, pathogen testing methods, and outbreak size can affect detection, investigation, and reporting (*4*). However, few methods are available to estimate the extent of outbreak underdetection and underreporting.

Outbreaks can be considered natural occurrences with a mathematical relationship between frequency and size. Several studies have used a power law distribution, where one variable is proportional to the power of another, to help describe disease outbreaks or transmission (*5*–*9*). We examined the mathematical relationship between foodborne outbreak frequency and size to estimate the number of expected outbreaks of different sizes, comparing power law, log-normal, and exponential distributions by using censored and complete data to clarify underdetection and underreporting.

## The Study

Local, state, and federal public health agencies in the United States identify and investigate foodborne outbreaks and report them to the Foodborne Disease Outbreak Surveillance System (FDOSS; https://www.cdc.gov/fdoss). In FDOSS, a foodborne outbreak is defined as >2 similar illnesses associated with a common food source. We used FDOSS data from 1998–2019 and defined outbreak size as the number of laboratory-confirmed cases. We also included outbreaks with >2 similar illnesses that had only 1 confirmed case. We evaluated the fit of power law, log-normal, and exponential distributions by applying the Kolmogorov-Smirnov (KS) statistic (*10*) to the number of outbreaks by size.

We estimated medians and 90% credible intervals (CrIs) for the minimum threshold, slope, and difference between expected and actual outbreak frequency by bootstrapping 5,000 random samples with replacement from the dataset of all outbreaks of the same size. We defined outbreaks of <10 confirmed cases as small and outbreaks of >100 confirmed cases as large. We conducted all analyses in R (The R Foundation for Statistical Computing, https://www.r-project.org) by using the poweRlaw package version 0.70.6 (*11*). We provide additional methods and R script (Appendix 1) and the dataset used (Appendix 2).

**Figure 1:**
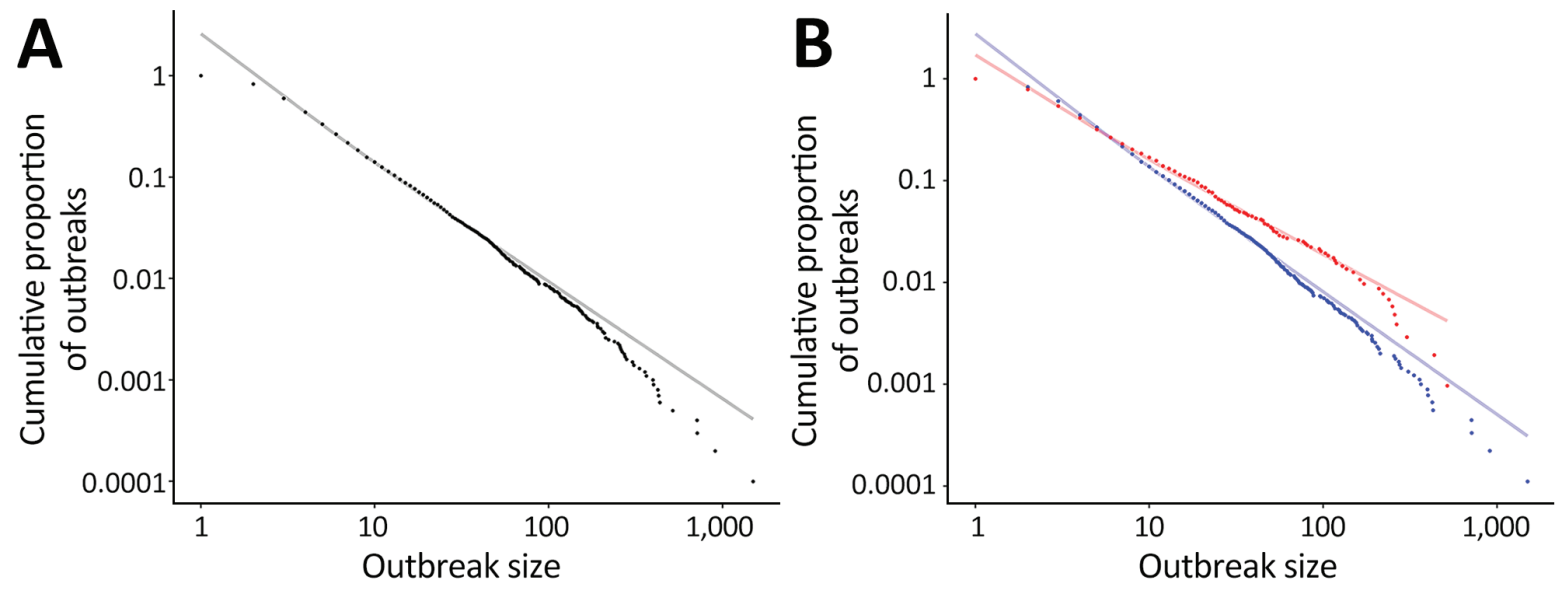
Log-log scale of foodborne outbreak size versus frequency from a power law for estimating underdetection of foodborne disease outbreaks, United States. A) Actual (black points) versus expected from the power law distribution (gray line) 1998–2017; B) actual (blue points) versus expected (light blue line) 1998–2019 and actual (red points) versus expected (light red line) 2018–2019. Estimates for the difference between the number of expected and actual small (<10 cases) and large (>100 cases) outbreaks were calculated by the sum of the differences between each of the relevant actual points and the expected line at the same x-value. Annual estimates were then calculated by dividing the number of years represented.

During 1998–2019, a total of 10,026 foodborne outbreaks were reported in the United States, ranging from 1­ to 1,500 laboratory-confirmed cases. The data appeared linear on a log-log scale, consistent with a power law distribution (Figure 1, panel A). We rejected the exponential and log-normal distributions because they fit poorly based on the KS statistic (exponential 0.109, p<0.001; log-normal 0.0101, p<0.001). The power law distribution fit the data (KS = 0.00985, p = 0.15).

**Figure 2:**
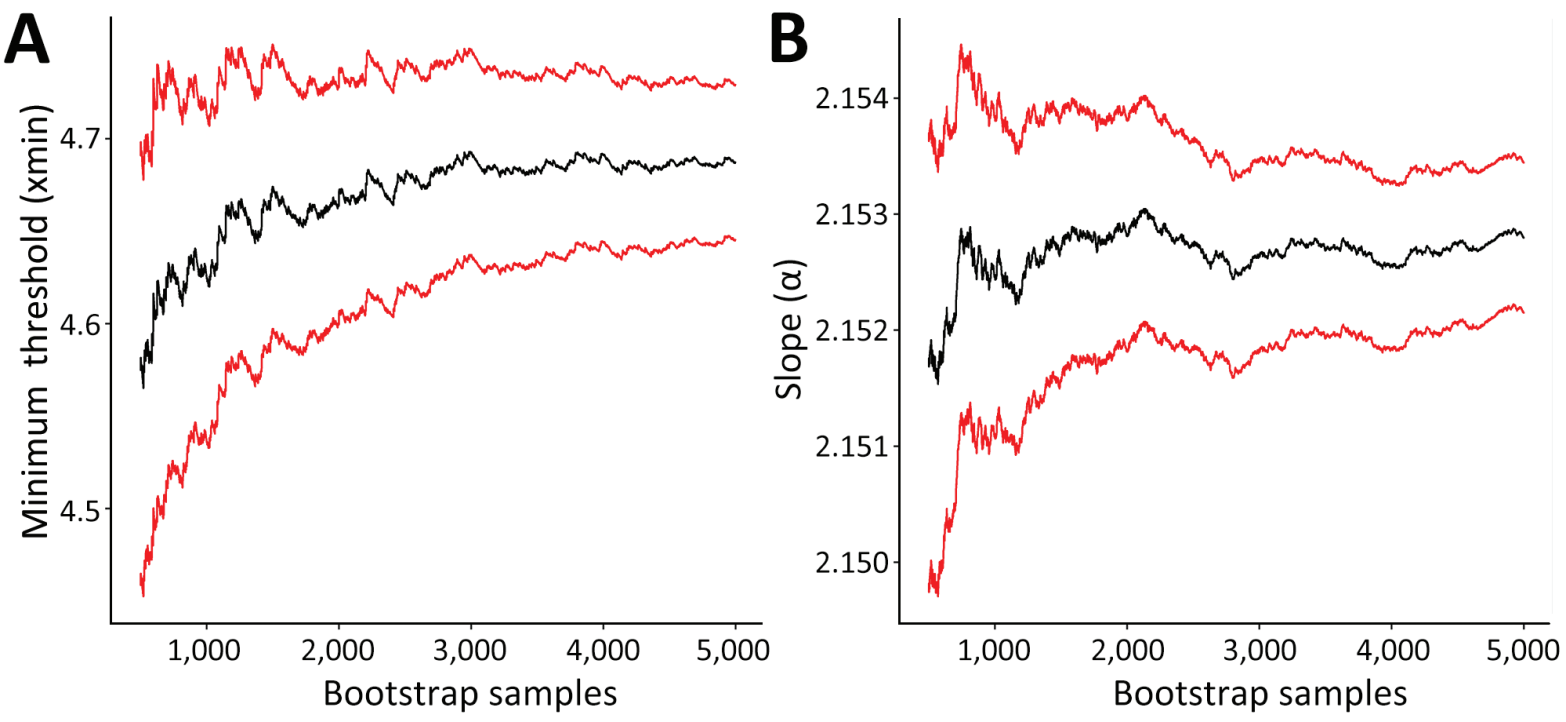
Parameter estimates from a power law for estimating underdetection of foodborne disease outbreaks, United States. Graphs display distribution of foodborne outbreak size and frequency for the minimum threshold (A) and slope (B) for outbreaks during 1998–2019. Black lines represent bootstrapped parameter estimate; red lines represent 90% credible intervals.

Foodborne outbreaks with >4 (90% CrI 4–8) cases followed a power law distribution of α = 2.15 (90% CrI 2.12–2.19) (Figure 2). We estimated 718 (90% CrI 594–783) fewer than expected small outbreaks and 0.4 (90% CrI −0.07–0.9) fewer than expected large outbreaks occurred annually, representing 841 (90% CrI 669–932) fewer than expected small outbreak-associated illnesses and 574 (90% CrI 325–871) fewer than expected large outbreak-associated illnesses.

By 2018, most US public health laboratories were using whole-genome sequencing (WGS) to subtype some bacteria that cause foodborne illness, including *Salmonella enterica*, *Escherichia coli*, and *Listeria monocytogenes*. WGS has helped public health practitioners detect more outbreaks and determine the food or other source while outbreaks are still small (*12*).

A power law distribution fit the outbreak data for both the 1998–2017 (8,993 outbreaks; KS = 0.00949, p = 0.37) and the 2018–2019 (1,033 outbreaks; KS = 0.0211, p = 0.43) periods (Figure 1, panel B). The minimum threshold was >5 cases (90% CrI 4–9) and α = 2.20 (90% CrI 2.16–2.25) during 1998–2017, compared with a minimum threshold of >3 cases (90% CrI 2–6) and α = 1.91 (90% CrI 1.83–2.00) during 2018–2019. We estimate 788 (90% CrI 665–888) fewer than expected small outbreaks and 0.4 (90% CrI −0.06 to 0.9) fewer than expected large outbreaks were identified annually during 1998–2017, compared with 365 (90% CrI 277–475) fewer than expected small outbreaks and 1 (90% CrI −3 to 2) more than expected large outbreak annually during 2018–2019.

## Conclusions

We found that foodborne disease outbreak data fit a power law distribution. On the basis of that finding, we quantified the unobserved burden of foodborne outbreaks in the United States during 1998–2019, predicting that 718 fewer than expected small outbreaks are detected, investigated, and reported every year and 1 fewer than expected large outbreak was detected and reported about every 3 years. Detection and reporting of foodborne outbreaks have improved; during 2018–2019, we estimate that underreporting of small outbreaks decreased by 54% (365/year) compared with 1998–2017 (788/year). The power law distribution quantifies improvements in detection and reporting, which could in part be explained by WGS.

Many factors affect outbreak and case detection, investigation, and reporting, including whether the outbreak is caused by a common molecular strain, how many persons ate the contaminated food, clinical manifestations, care-seeking, diagnostic testing, and laboratory or health department outbreak investigation and response capacity. Natural limitations to outbreak size are also likely, including the geographic distribution of a contaminated food product, food safety policies that control contamination in the food system, and product recalls or other disease control efforts that end large outbreaks before natural limitations are reached.

Power law distribution parameters should be stable over time, but changes in the slope or minimum threshold or deviations from the estimated power law might indicate perturbations of concern. Understanding the different power law parameters that underlie outbreak size and frequency can also be useful for exploring how detection of foodborne outbreaks differs by pathogen or food vehicle. In addition, those parameter changes can reflect public health interventions.

The power law distribution has applications beyond foodborne outbreaks and has been applied to COVID-19, measles, and gonorrhea (*5*–*9*). By predicting outbreak frequency and the extent of underdetection, we can plan outbreak response needs for routine and surge scenarios, assess the effects of outbreak prevention efforts, and improve estimates of the proportion of illnesses that are outbreak-associated versus sporadic.

A limitation of this analysis is that failure to statistically reject the power law distribution does not ensure that the data follow a power law. The KS statistic also might miss systematic patterns that differ between distributions because it uses only the largest difference. However, we used a hypothesis-driven rationale to censor data by establishing a minimum threshold, tested alternative distributions, and characterized uncertainty by using the bootstrap. Another limitation is that we only include reported outbreaks with laboratory confirmed cases, which could underestimate cases but also reduces variation from comparing across multiple types of outbreaks. Laboratory-confirmed cases also could be an underestimate for the largest outbreaks because public health laboratories might run out of resources to subtype patient samples or be faced with other constraints due to the overwhelming size of the outbreak.

In conclusion, we used the power law distribution on foodborne disease outbreak data to quantify underdetection and how foodborne disease reporting has improved. The improvement in underdetection during 2018–2019 could in part be explained by improved detection or investigation from the implementation of WGS. The power law distribution can be used to assess the impact of past and future public health interventions and as a tool for resource planning.

Appendix 1 R code used for a power law for estimating underdetection of foodborne disease outbreaks, United States.

Appendix 2 Data set used for a power law for estimating underdetection of foodborne disease outbreaks, United States.
