# Supplementary material for: Power Law for Estimating Underdetection of Foodborne Disease Outbreaks, United States
Source: Emerg Infect Dis. 2024 Feb;30(2):337–40. doi: 10.3201/eid3002.230342 (PMC10826756; doi:10.3201/eid3002.230342)
Supplement: Appendix 1 — R code used for a power law for estimating underdetection of foodborne disease outbreaks, United States. [file 23-0342-Techapp-s1.pdf]

Article DOI: https://doi.org/10.3201/eid3002.230342

EID cannot ensure accessibility for supplementary materials supplied by authors. Readers who have difficulty accessing supplementary content should contact the authors for assistance.

Power Law for Estimating Underdetection of Foodborne Disease Outbreaks, United States

Appendix 1

Methods

The R (The R Foundation for Statistical Computing, https://www.r-project.org) script used in this analysis is below. The script evaluates the fit of an exponential, log-normal and power law distribution and estimates the median and 90% credible interval for minimum threshold, slope, and difference between expected and actual frequency based on the power law distribution. The estimates for the difference between the number of expected and actual small (<10 cases) and large (>100 cases) outbreaks were calculated by the sum of the differences between each of the relevant actual points and the expected line at the same x-value. Annual estimates are then calculated by dividing the number of years represented.

#The following is the R script used in: Ford L, Self JL, Wong KK, Hoekstra RM, Tauxe RV, Rose EB, Bruce BB. Power Law for Estimating Underdetection of Foodborne Disease Outbreaks, United States . Last modified in 2023.

```
library(tidyverse)
library(poweRlaw)
library(readxl)
library(colorspace)
library(ggpubr)
##########################################################################################
##
##
```

```
## Prepare data
##
#####################################################################################
##
read_excel(“FORD_20220531_forrelease.xlsx,”
sheet = “FORD_20220531_forreview2”) %>%
filter(ConfirmedPrimary ! = 0 and yearfirstill ! = 2020) %>%
.$ConfirmedPrimary %>%
na.omit %>%
as.vector ->
d
#####################################################################################
##
##
## Explore distribution fits
##
#####################################################################################
##
## Discrete exponential distribution
dexp <- disexp$new(d)
dexp$setPars(estimate_pars(dexp))
dexp_p < - bootstrap_p(dexp, xmins = 1:100, no_of_sims = 5000, threads = 4)
plot(dexp_p)
dexp_p$p
## Log normal distribution
dln <- dislnorm$new(d)
dln$setPars(estimate_pars(dln))
dln_p < - bootstrap_p(dln, xmins = 1:100, no_of_sims = 5000, threads = 4)
plot(dln_p)
dln_p$p
## Power law distribution with cutoff
dplco <- displ$new(d)
dplco$setXmin(estimate_xmin(dplco))
```

```
dplco$setPars(estimate_pars(dplco))
dplco_p < - bootstrap_p(dplco, xmins = 1:20, no_of_sims = 5000, threads = 4)
plot(dplco_p)
dplco_p$p
quantile(dplco_p$bootstraps$xmin[1:5000], probs = c(0.05, 0.5, 0.95))
quantile(dplco_p$bootstraps$pars[1:5000], probs = c(0.05, 0.5, 0.95))
###################################################################################
##
##
## Estimate the difference between actual and expected from
## power law distribution overall (1998–2019)
##
###################################################################################
##
# properly aligned cumsum for our purposes
fixcumsum <- function(x) {
x <- rev(x)
for (i in head(seq_along(x), −1)) {
if (is.na(x[i + 1])) {
x[i + 1] <- x[i]
}
}
rev(x)
}
# apply the bootstrap parameters to the distribution object and return expected versus actual
uo2 <- function(dis, α, altxmin) {
oldalpha <- dis$getPars()
dis$setPars(α)
oldxmin <- dis$getXmin()
dis$setXmin(altxmin)
xmin <- altxmin
vals <- dis$internal$values
lvals <- log10(vals)
```

```
cumn <- dis$internal$cum_n
lcumn <- log10(cumn)
plot(lvals, lcumn, pch = 20)
yhat <- dist_cdf(dis, tail(vals, -(xmin - 1)), FALSE)
dis$setXmin(1)
scale <- dist_data_cdf(dis, FALSE)[xmin]
cumn <- dis$internal$cum_n[1] * yhat * scale
lcumn <- log10(cumn)
dis$setXmin(oldxmin)
slope <- diff(head(lcumn, 2)) / diff(tail(lvals, -(xmin - 1)))[1]
icept <- lcumn[1] - slope * log10(xmin)
lcumn <- c(slope * lvals[1:(xmin - 1)] + icept, lcumn)
cumn <- c(10 ^ lcumn[1:(xmin - 1)], cumn)
lines(lvals, lcumn, col = “red”)
# extrapolate to y = 0 using CDF
slope2 <- diff(tail(lcumn, 2)) / diff(tail(lvals))
icept2 <- tail(lcumn, 1) - slope2 * max(lvals)
maxx <- c(lvals, -icept2 / slope2)
xs <- min(vals):max(10 ^ maxx)
lxs <- log10(xs)
lyhats <- approx(c(lvals, max(lxs)), c(lcumn, 0), lxs)$y
yhats <- 10 ^ lyhats
out <- data.frame(xs, lxs, lyhats, yhats)
out <- merge(out, data.frame(xs = dis$internal$values,
yobs = dis$internal$cum_n),
all.x = TRUE)
out$yobs <- fixcumsum(out$yobs)
out$yobs[is.na(out$yobs)] <- 0
out$ydiff <- c(-diff(out$yhats) - -diff(out$yobs),
tail(out$yhats, 1) - tail(out$yobs, 1))
out$ndiff <- out$xs * out$ydiff
dis$setPars(oldalpha)
out
```

```
}
## function to calculate difference in actual versus expected for small and large
## outbreaks
small_big <- function(dis, dis_p) {
out <- list()
boot_slope <- dis_p$bootstraps$pars
boot_xmin <- dis_p$bootstraps$xmin
for (i in 1:5000) {
o <- uo2(dis, boot_slope[i], boot_xmin[i])
# includes confirmed ill of size = 1 to 9
smallobs <- with(o, sum(ydiff[xs <10]))
smallills <- with(o, sum(ndiff[xs <10]))
# includes confirmed ill of size 100 or larger
bigobs <- with(head(o, −1), sum(ydiff[xs >100]))
bigills <- with(head(o, −1), sum(ndiff[xs >100]))
out$diffs$small_obs <- c(out$diffs$small_obs, smallobs)
out$diffs$small_ills <- c(out$diffs$small_ills, smallills)
out$diffs$big_obs <- c(out$diffs$big_obs, bigobs)
out$diffs$big_ills <- c(out$diffs$big_ills, bigills)
}
out$summary <- lapply(out$diffs, quantile, probs = c(0.05, 0.5, 0.95))
out
}
o_small_big <- small_big(dplco, dplco_p)
################################################################################
##
##
## Fit separate power law distributions to 1998–2017 and 2018–2019 and compare
##
################################################################################
##
### 1998–2017
read_excel("FORD_20220531_forrelease.xlsx,"
```

```
sheet = "FORD_20220531_forreview2") %>%
filter(ConfirmedPrimary ! = 0 and yearfirstill <2018) %>%
.$ConfirmedPrimary %>%
na.omit %>%
as.vector ->
bwgs
bwgsplco <- displ$new(bwgs)
bwgsplco$setXmin(estimate_xmin(bwgsplco))
bwgsplco$setPars(estimate_pars(bwgsplco))
bwgsplco_p < - bootstrap_p(bwgsplco, xmins = 1:20, no_of_sims = 5000, threads = 4)
plot(bwgsplco_p)
bwgsplco_p$p
quantile(bwgsplco_p$bootstraps$xmin[1:5000], probs = c(0.05, 0.5, 0.95))
quantile(bwgsplco_p$bootstraps$pars[1:5000], probs = c(0.05, 0.5, 0.95))
boot_slope_bwgs <- bwgsplco_p$bootstraps$pars
boot_xmin_bwgs <- bwgsplco_p$bootstraps$xmin
b_small_big <- small_big(bwgsplco, bwgsplco_p)
## 2018–2019
read_excel("FORD_20220531_forrelease.xlsx,"
sheet = "FORD_20220531_forreview2") %>%
filter(ConfirmedPrimary ! = 0 &
(yearfirstill > = 2018 and yearfirstill <2020)) %>%
.$ConfirmedPrimary %>%
na.omit %>%
as.vector ->
awgs
awgsplco <- displ$new(awgs)
awgsplco$setXmin(estimate_xmin(awgsplco))
awgsplco$setPars(estimate_pars(awgsplco))
awgsplco_p < - bootstrap_p(awgsplco, xmins = 1:20, no_of_sims = 5000, threads = 4)
plot(awgsplco_p)
awgsplco_p$p
awgsplco_p$bootstraps
```

```
quantile(awgsplco_p$bootstraps$xmin[1:5000], probs = c(0.05, 0.5, 0.95))
quantile(awgsplco_p$bootstraps$pars[1:5000], probs = c(0.05, 0.5, 0.95))
boot_slope_awgs <- awgsplco_p$bootstraps$pars
boot_xmin_awgs <- awgsplco_p$bootstraps$xmin
a_small_big <- small_big(awgsplco, awgsplco_p)
########################################################################################
##
##
## Figures
##
########################################################################################
##
## Figure 1
options(scipen = 100000)
# function to generate consistent plot data
plot_data <- function(dis) {
xmin <- dis$getXmin()
uo2(dis, dis$getPars(), xmin) %>%
filter(xs %in% dis$internal$values) %>%
mutate(yobs = yobs / yobs[1], yhats = yhats / yhats[xmin] * yobs[xmin])
}
# function to generate a consistent power law plot
ggpl <- function(data) {
if (is.null(data$grp)) data$grp <- “A”
ggplot(data, aes(x = xs, y = yhats, group = grp, color = grp)) +
geom_line(linewidth = 1, α = 0.25) +
geom_point(aes(y = yobs), size = 0.5) +
scale_x_log10(name = “Outbreak size”) +
scale_y_log10(name = “Cumulative proportion of outbreaks,”
breaks = 10 ^ (−4:0),
labels = 10 ^ (−4:0)) +
theme_bw()+
theme(panel.grid.major = element_blank(), panel.grid.minor = element_blank())
```

```
}
ggpl(plot_data(dplco)) +
ggtitle("A.") +
scale_color_manual(values = "black") ->
all_years
plot_data(bwgsplco) %>%
mutate(grp = "1998-2017") %>%
bind_rows(plot_data(awgsplco) %>%
mutate(grp = "2018-2019")) %>%
ggpl() +
ggtitle("B.") +
scale_color_manual(values = c("blue," "red")) ->
comb
tiff(filename = "Figure 1.tiff," height = 5, width = 12,
units = "in," res = 600)
ggarrange(all_years, comb, ncol = 2, legend = "none")
dev.off()
## Figure 2
plot_data2 <- function(dpl) {
dplco_p$bootstraps %>%
select(xmin, pars) %>%
map(poweRlaw:::get_cum_summary) %>%
imap(~ mutate(.x, var = .y)) %>%
map(~ select(., x, var, m, m_up, m_low) %>%
pivot_longer(-c(var, x))) %>%
bind_rows %>%
mutate(var = ifelse(var =  = "pars," "alpha," "X_min"),
var = recode(var, "alpha" = "Slope (\u03b1),"
"X_min" = "Minimum Threshold (xmin)"))
}
tiff(filename = "Figure 2.tiff," height = 5, width = 10, units = "in," res = 600)
plot_data2(dplco_p) %>%
ggplot(aes(x = x, y = value, color = name)) +
```

```
geom_line() +
facet_wrap(~ var, scales = “free”) +
scale_color_manual(values = c(“black,” “red,” “red”)) +
guides(color = “none”) +
xlab(“Bootstrap samples”) +
theme_bw() +
theme(axis.title.y = element_blank(), panel.grid.major = element_blank(), panel.grid.minor =
element_blank())
dev.off()
```
